# Relationship between betacoronaviruses and the endocrine system: a new key to understand the COVID-19 pandemic—A comprehensive review

**DOI:** 10.1007/s40618-020-01486-0

**Published:** 2021-02-13

**Authors:** T. Piticchio, R. Le Moli, D. Tumino, F. Frasca

**Affiliations:** Endocrinology Section, Department of Clinical and Experimental Medicine, Garibaldi Nesima Hospital, University of Catania, Via Palermo 636, 95122 Catania, Italy

**Keywords:** SARS, COVID-19, Thyroid, Pituitary, Adrenal gland, Endocrine system

## Abstract

**Background:**

A new harmful respiratory disease, called COVID-19 emerged in China in December 2019 due to the infection of a novel coronavirus, called SARS-Coronavirus 2 (SARS-CoV-2), which belongs to the betacoronavirus genus, including SARS-CoV-1 and MERS-CoV. SARS-CoV-2 shares almost 80% of the genome with SARS-CoV-1 and 50% with MERS-CoV. Moreover, SARS-CoV-2 proteins share a high degree of homology (approximately 95%) with SARS-CoV-1 proteins. Hence, the mechanisms of SARS-Cov-1 and SARS-Cov-2 infection are similar and occur via binding to ACE2 protein, which is widely distributed in the human body, with a predominant expression in endocrine tissues including testis, thyroid, adrenal and pituitary.

**Purpose:**

On the basis of expression pattern of the ACE2 protein among different tissues, similarity between SARS-Cov-1 and SARS-Cov-2 and the pathophysiology of COVID-19 disease, we aimed at discussing, after almost one-year pandemic, about the relationships between COVID-19 infection and the endocrine system. First, we discussed the potential effect of hormones on the susceptibility to COVID-19 infection; second, we examined the evidences regarding the effect of COVID-19 on the endocrine system. When data were available, a comparative discussion between SARS and COVID-19 effects was also performed.

**Methods:**

A comprehensive literature search within Pubmed was performed. This review has been conducted according to the PRISMA statements.

**Results:**

Among 450, 100 articles were selected. Tissue and vascular damages have been shown on thyroid, adrenal, testis and pituitary glands, with multiple alterations of endocrine function.

**Conclusion:**

Hormones may affect patient susceptibility to COVID-19 infection but evidences regarding therapeutic implication of these findings are still missing. SARS and COVID-19 may affect endocrine glands and their dense vascularization, impairing endocrine system function. A possible damage of endocrine system in COVID-19 patients should be investigated in both COVID-19 acute phase and recovery to identify both early and late endocrine complications that may be important for patient’s prognosis and well-being after COVID-19 infection.

## Introduction

During December 2019, a new fatal respiratory disease emerged in Wuhan, Hubei province, China [1–3]. This disease can cause atypical pneumonia and acute respiratory distress syndrome with a relatively high risk of death for patients [1, 4]. The causative agent of this lung disease is a novel coronavirus, called SARS-coronavirus 2 (SARS-CoV-2), which is closely related to SARS-CoV-1 [3]. In March 2020, because of the rapid and wide spread of the infection around the world, WHO declared pandemic for this new disease and named it as COVID-19.

SARS-Cov-2 virus belongs to the betacoronavirus genus including SARS-CoV-1 and MERS-CoV pathogens that caused epidemics in 2002 and 2013, respectively [5]. All authors agree with the assumption that SARS-CoV-2 alert depends on the its great ability to spread, with R0 about 3.8 (1.4–6.49) [6], rather than on the mortality rate per se, which stands at around 2.4% according to WHO report in November, 22, 2020 [7] (at variance with MERS with R0 < 1 and mortality rate of 34.4% and SARS with R0 of approximately 1.8 and mortality rate of 10%) [8]. Human-to-human transmission occurs by droplet and contact routes, although airborne, fecal or intrauterine transmission may be also considered. The mechanism of infection is similar in both SARS-CoV-1 and SARS-CoV-2 and occurs via virus binding to protein ACE2 [9] and the intervention of the TMPRSS2 protease and, at lesser extent, of cathepsin B (CTSB) and L (CTSL) [10], which are widely distributed in human body, with a relevant expression in endocrine tissues including testicle, thyroid, adrenal and pituitary [11, 12]. On the other hand, already at the onset of SARS, MERS and COVID-19 epidemics, it was evident that hormonal and metabolic conditions may influence the outcome of viral disease. For instance, diabetes is an important risk factor for poor prognosis and mortality [13, 14]. In respect to COVID-19 infection, current data indicate that elderly, hypertension, obesity and diabetes are important risk factors for mortality [15, 16]. More importantly, Cushing’s Syndrome (including most of the above-mentioned risk) and pre-existent adrenal insufficiency (impairing patient recovery capabilities) are major determinants of COVID-19 disease outcome [17–19]. Hence, the strict relationship between the COVID-19 and the endocrine system raised the interest of the endocrinologists for this emerging pandemic. For these reasons, international scientific societies of endocrinology and Italian Society of Endocrinology have given important contributions to the literature with the aim of suggesting the optimal management of the endocrine high-risk patients during the COVID-19 pandemic [17–20]. Moreover, on the basis of tissue expression pattern of the proteins ACE2 and TMPRSS2, a twelve-month experience of COVID-19 disease and similarities between SARS-CoV-1 and SARS-CoV-2, this review will also try to summarize possible short- and long-term alterations of the endocrine system in subjects affected by COVID-19.

## Methods

This review was conducted according to the Preferred Reporting Items for Systematic Reviews and Meta-Analyses (PRISMA) statements. A comprehensive literature search within Pubmed was performed from outbreak of the pandemic until 18 November 2020. Search terms included “Covid-19”, “SARS-Cov-2”, “SARS-Cov”, “MERS-Cov”, “Endocrine system”, “Hypotalamus”, “Pituitary”, “Thyroid”, “Adrenal Gland”, “Testis”, “ACE2”, “ACE2 expression”, “TMPRSS2”, “TMPRSS2 expression”, “COVID-19 treatments” and “COVID-19 drugs”. This search strategy was enhanced by tracking citations of articles included in Google Scholar.

**Fig. 1:**
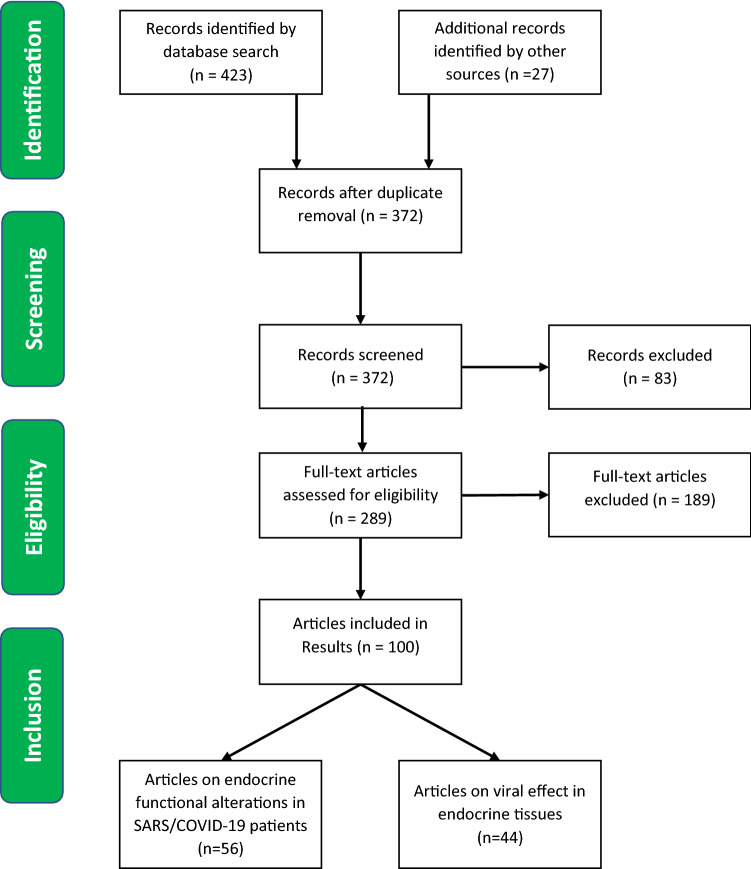
PRISMA flow diagram for paper selection

Given the limited number of works available, we have taken into account all papers describing in SARS and COVID-19 patients any pathological or functional alteration in hypothalamus/pituitary axis, thyroid, adrenal gland, ovary and testis. We also included studies that detected the presence of SARS-COV-1 and SARS-COV-2 in endocrine glands by in situ hybridization, electron microscopy and RT-PCR. Furthermore, the research focused on: similarities between SARS-CoV-2 and other beta-coronaviridae; ACE2 and TMPRSS2 expression in endocrine tissues; ACE2 expression and COVID-19 treatments; coronavirus infections and potential damage systems to endocrine glands and hormonal regulation of TMPRSS2 expression. In this regard, all types of studies were considered, including studies on gene databases. Two investigators screened title/abstracts for potential eligibility. Disagreements were resolved through consensus. Due to fragmentary and heterogeneous results, the small number of papers (especially about SARS) and the recent onset of COVID-19 pandemic, we conducted a synthesis of all the available data (Fig. 1).

## Results and discussion

### Systematic search results

The global search returned 450 titles, and 372 were available after the removal of duplicates. We selected 289 papers to assess the full-text. Subsequently, we excluded 189 of the retrieved articles. A total of 100 papers were included (Tables 1 and 2).

**Table 1:** List of selected articles containing evidences on endocrine functional alterations in SARS and COVID-19 patients

Endocrine links to SARS and COVID-19 pathophysiology	Reference number	Author (Year)
Similarities between SARS-CoV-2 and other beta-coronaviridae	[5]	Chen Y (2020)
	[10]	Hoffmann M (2020)
	[21]	Siddell SG (2005)
	[22]	Cavanagh D (1995)
	[23]	Kandeel M (2018)
	[24]	Risco C (1996)
	[25]	Ruch T (2012)
	[26]	Neuman BW (2011)
	[27]	Chan JFW (2020)
	[28]	Rabaan AA (2020)
	[29]	Xu J (2020)
	[30]	Li W (2003)
	[31]	Matsuyama S (2010)
	[32]	Shulla A (2011)
	[33]	Zhang C (2020)
	[34]	Zhou P (2020)
	[35]	Fehr AR (2015)
ACE2 and TMPRSS2 expression in endocrine tissues	[11]	Chen Y (2020)
	[12]	Lazartigues E (2020)
	[63]	Hamming I (2004)
	[64]	Chi M (2020)
	[100]	Reis FM (2011)
	[101]	Jing Y (2020)
	[102]	Goad J (2020)
	[103]	Wang Z (2020)
	[104]	Fan C (2020)
ACE2 expression and COVID-19 treatments	[36]	Malek Mahdavi A (2020)
	[37]	Xiang Z (2020)
	[38]	Young MJ (2020)
	[39]	Hanff TC (2020)
	[40]	Marshall RP (2000)
	[41]	Marshall RP (2004)
	[42]	Mastruzzo C (2002)
	[43]	Okada M (2009)
	[44]	Wang R (1999)
	[45]	Keidar S (2007)
	[46]	Mizuiri S (2015)
	[47]	Imai Y (2008)
	[48]	Xu J (2017)
	[49]	Panarese A (2020)
Coronavirus infections and potential damage systems to endocrine glands	[65]	Ding Y (2003)
	[66]	Guo Y (2008)
	[67]	Yao XH (2020)
	[77]	Conti P (2020)
	[99]	Giannis D (2020)
Hormonal regulation of TMPRSS2 expression and its role in susceptibility to COVID-19 infection	[50]	Jin J (2020)
	[51]	Bertram S (2013)
	[52]	Asselta R (2020)
	[53]	Li MY (2020)
	[54]	Stopsack KH (2020)
	[55]	Thebault R (2020)
	[56]	Bennett CL (2002)
	[57]	Wambier CV (2020)
	[58]	Montopoli M (2020)
	[59]	Chen J (2020)
	[60]	Dasinger JH (2016)
	[61]	Mishra JS (2016)
	[62]	Klein SL (2012)

**Table 2:** List of selected articles containing evidences of SARS, COVID-19 infection and endocrine tissue damage

Endocrine gland/system	Reference number	Author (Year)	Infection	Kind of study
Hypothalamus/Pituitary	[68]	Zhang QL (2003)	SARS	Pathology
	[69]	Ding Y (2004)	SARS	Pathology
	[70]	Gu J (2005)	SARS	Pathology
	[71]	Wei L (2010)	SARS	Pathology
	[72]	Ye YX (2004)	SARS	Clinical
	[73]	Wang W (2003)	SARS	Clinical
	[74]	Leow MK (2005)	SARS	Clinical
	[75]	Zhou L (2020)	COVID-19	Case report
	[76]	Li T (2020)	COVID-19	Clinical
Thyroid	[67]	Yao XH (2020)	COVID-19	Pathology
	[70]	Gu J (2005)	SARS	Pathology
	[74]	Leow MK (2005)	SARS	Clinical
	[76]	Li T (2020)	COVID-19	Clinical
	[78]	Wei L (2007)	SARS	Pathology
	[79]	Wang W (2003)	SARS	Clinical
	[82]	Chen M (2020)	COVID-19	Clinical
	[83]	Chen T (2020)	COVID-19	Clinical
	[84]	Gao W (2020)	COVID-19	Clinical
	[85]	Lui DTW (2020)	COVID-19	Clinical
	[86]	Khoo B (2020)	COVID-19	Clinical
	[87]	Brancatella A (2020)	COVID-19	Case report
	[88]	Ippolito S (2020)	COVID-19	Case report
	[89]	Asfuroglu K E (2020)	COVID-19	Case report
	[90]	Ruggeri RM (2020)	COVID-19	Case report
	[91]	Brancatella A (2020)	COVID-19	Case report
	[92]	Lania A (2020)	COVID-19	Clinical
	[93]	Muller I (2020)	COVID-19	Clinical
Adrenal gland	[65]	Ding Y (2003)	SARS	Pathology
	[68]	Zhang QL (2003)	SARS	Pathology
	[70]	Gu J (2005)	SARS	Pathology
	[94]	Zinserling VA (2020)	COVID-19	Pathology
	[95]	Freire S M (2020)	COVID-19	Pathology
	[96]	Iuga AC (2020)	COVID-19	Pathology
	[97]	Frankel M (2020)	COVID-19	Case report
	[98]	Álvarez-T J (2020)	COVID-19	Case report
Ovary	[69]	Ding Y (2004)	SARS	Pathology
Testis	[69]	Ding Y (2004)	SARS	Pathology
	[70]	Gu J (2005)	SARS	Pathology
	[105]	Xu J (2006)	SARS	Pathology
	[106]	Wang DW (2003)	SARS	Pathology
	[107]	Zhao JM (2003)	SARS	Pathology
	[108]	Yang M (2020)	COVID-19	Pathology
	[109]	Achua JK (2020)	COVID-19	Pathology
	[110]	Li H (2020)	COVID-19	Clinical/Pathology
	[111]	Ma L (2020)	COVID-19	Clinical
	[112]	Pan F (2020)	COVID-19	Clinical
	[113]	Song C (2020)	COVID-19	Clinical
	[114]	Paoli D (2020)	COVID-19	Case report
	[115]	Holtmann N (2020)	COVID-19	Clinical
	[116]	Li D (2020)	COVID-19	Clinical
	[117]	Vishvkarma R (2020)	COVID-19	Sistematic review
	[118]	Corona G (2020)	COVID-19	Position statement

### Similarities between SARS-CoV-2 and other beta-coronaviridae

Genome sequence analysis indicated that SARS-CoV-2 belongs to the betacoronavirus genus, including SARS-CoV-1 and MERS-CoV [5]. These coronaviridae are enveloped, positive-stranded RNA viruses with a large genome approaching 30 kb and including four structural proteins, namely spike (S), nucleocapsid (N) envelope (E), and membrane (M) [21]. The S protein is responsible for virus attachment to the receptor and fusion to the cell membrane [22, 23]. The N protein interacts with the viral RNA to form the ribonucleoprotein [24]. The E protein helps virion assembly and includes ion channel actions [25]; the M protein participates to the assembly of new virus particles [26]. SARS-CoV-2 shares almost 80% of the genome with SARS-CoV-1 [27] and 50% with MERS-CoV [28]. Moreover, SARS-CoV-2 proteins share a high degree of homology (approximately 95%) with the SARS-CoV-1 proteins [29]. Among the various viral proteins, the S protein is the most important to infect host cells as it facilitates viral entry into target cells. Entry depends on binding of the surface unit, S1 of the S protein to a cellular receptor, which facilitates viral attachment to the surface of target cells. In addition, entry requires S protein priming by cellular proteases, which entail S protein cleavage at the S1/S2 and the S2′ site and allow fusion of viral and cellular membranes, a process driven by the S2 subunit. SARS-S engages angiotensin-converting enzyme 2 (ACE2) as the entry receptor [30] and employs the cellular serine protease TMPRSS2 for S protein priming or, at lesser extent, cathepsin B (CTSB) and L (CTSL) [10, 31–33]. SARS-S and SARS-2-S share 76% amino acid identity and SARS-2-S protein exploits ACE2 for entry with the subsequent intervention of TMPRSS2 [10, 34]. These data suggest that SARS-CoV-2 shares the similar target cells with SARS-CoV-1 [10]. On the other hand, MERS-CoV, binds to dipeptidyl-peptidase 4 (DPP4) to enter human cells [35].

### Effect of hormones on human susceptibility to coronavirus infection

#### Glucocorticoid and vitamin D

Some authors wondered whether hormonal treatment of COVID-19 patients may affect ACE2 expression in tissues and, as a consequence, viral spread. To date, some evidences indicate that both vitamin D and glucocorticoids may increase the systemic expression of ACE2 receptors [36–38].

However, according to these data, this increase in potential sites for viral entry is only apparently disadvantageous, rather it is essential to preserve the integrity of the parenchyma of various tissues, including endocrine glands. In fact, the binding of SARS-CoV-2 with human ACE2 reduces its expression, thereby causing a rebound generation of Angiontensin II (Ang II) via the ACE enzyme [36, 39]. In turn, Ang II overproduction may stimulate cell growth, fibrosis, epithelial cell apoptosis, generation of reactive oxygen species and the release of proinflammatory cytokines, thereby enhancing the detrimental effect of infection [40–44]. On the other hand, ACE2 cleaves Ang II into angiotensin 1–7 [Ang-(1–7)] which acts as a vasodilator, reducing blood pressure [45] by stimulating nitric oxide synthase (NOS) [46]. By antagonizing the Ang II actions, Ang-(1–7) improves fasting glycemia and glucose tolerance, stimulates β-cell proliferation, prevents β-cell apoptosis and protects kidney function [12, 46]. For all these mechanisms, ACE/ACE2 ratio is believed to have an important impact on various diseases including diabetes, hypertension, nephropathy and also on the prognosis of COVID-19 patients [12, 47–49].

#### Sex hormones

Although men and women have a similar susceptibility to SARS-CoV-2, men appear to be prone to a more severe disease and mortality in an age-independent manner [50]. Indeed, several evidences indicate that TMPRSS2 expression in human tissues is regulated by androgen receptor activity [51]. It is reasonable to suppose that a higher TMPRSS2 expression level in males may contribute to the more severe COVID-19 infection compared to women and prepubertal children. In this respect, one study analyzing genomic databases found a higher TMPRSS2 mRNA expression in lung samples from men than from women (*p* = 0.029), while ACE2 mRNA expression level was not significantly different [52]. This observation was confirmed by Li et al. that was not able to find any difference in ACE2 expression level between males and females, younger and older subjects [53]. However, further studies are needed to confirm whether this different expression of TMPRSS2 in males and females may influence the virus pathogenicity [54]. In addition, it has been hypothesized that CAG repeats of the androgen receptor gene, regulating sensitivity to androgens, may be also associated to COVID-19 disease severity. According to this hypothesis, androgen receptor genetic variants that have been associated with androgenetic alopecia, prostate cancer, benign prostatic hyperplasia and polycystic ovary syndrome may be associated to higher susceptibility to COVID-19 infection. In accordance with these findings, some authors reported a disproportionate mortality rate in African-American COVID-19 patients [55], which carry a shorter variant of the CAG repeat in the androgen receptor gene [56]. Indeed, men hospitalized for severe COVID-19 disease displayed a higher rate of androgenetic alopecia compared to the general population [57]. Finally, Montololi et al. described a protective effect of anti-androgen therapies for prostate cancer against SARS-CoV-2 infection [58]. In accordance with this hypothesis, several authors proposed anti-androgen treatment of exposed subjects to reduce disease severity. However, careful prospective clinical trials are needed to validate this strategy as adjuvant therapy for COVID-19.

Moreover, studies performed on both animal and human models suggest that androgen suppression results in increased ACE2 expression [59–61], while it is unclear whether this effect results in increased risk of severe infection or in a protective effect of ACE2 protein overexpression (as previously discussed). Moreover, androgens and estrogens may influence immune response against viral infection in an opposite way and adverse reaction rates to both vaccines and antiviral drug are consistently higher in females than males [62]. Hence, caution is necessary before designing pharmacological strategies on this issue.

### Effect of coronavirus on the endocrine system

#### ACE2 and TMPRSS2 expression in endocrine tissues

Studies on the distribution of the ACE2 protein in human tissues allowed the identification of the potential pathways of infection and suggested the pathogenetic implications of the infection of SARS-CoV-1 and SARS-CoV-2. These evidences indicated a maximal ACE2 expression in lung, small intestine enterocytes and a lower expression in testis, thyroid, adipose tissue, ovary and endothelia. Furthermore, ACE2 was detected in adrenals, prostate, pituitary and hypothalamus [11–13]. Lazartigues et al., confirmed the ACE2 and TMPRSS2 mRNA expression in endocrine tissue, both in males and females, reinforcing the hypothesis of an endocrine involvement during viral infection [12]. Finally, since TMPRSS2 has also been identified in extracellular vesicles [64], it is reasonable to suppose that this protease may reach other tissues beyond its expression sites, thereby contributing to spreading of SARS-CoV-2 infection.

#### Mechanisms of potential damage on endocrine glands induced by coronavirus infection

The ACE2 expression pattern is in agreement with the pathological studies performed in SARS-CoV-1- or SARS-CoV-2-infected patients, displaying a variable degree of damage in the endocrine tissues including direct cell damage due to viral entry and replication, vasculitis, arteriolar and venular thrombosis, hypoxic cell damage, consequent immune response and cytokine storm [65, 66].

In particular, thrombosis was more common in COVID-19 patients, in small vessels and also in extrapulmonary organs, rather than in SARS patients [67]. In general point of view, this specific pathogenetic effect of COVID-19 may affect highly vascularized organs, such as the endocrine glands and in particular those with a very dense vascular network including pituitary (Table 3).

**Table 3:** Explanatory summary of the results

Endocrine gland/system	Infection	Pathological features	Endocrine function
Thyroid	SARS	Derangement of the follicular architecture [78] High levels of apoptosis (by Tunel), in both follicular epithelium and in interfollicular region [78] Interfollicular fibrosis [78] Absence of calcitonin-positive cells [78]	FT3 and FT4 levels significantly lower than control group [79] FT3 and FT4 levels decreased, respectively, by 94% and 46%, during the acute phase of illness [79] FT3 and FT4 levels decreased, respectively, by 90% and 38% during the convalescence phase of illness [79]
	COVID-19	No abnormalities in thyroid follicular cells [67] Interstitial lymphocytic infiltration [67]	During recovery, TSH and FT3 levels were significantly lower in patients than in healthy subjects [82] Decrease in TSH and TT3 or FT3 levels positively correlated to the infection severity [76, 82–85] Mild reductions of TSH and FT4 in admission to hospital [86] Normalization of thyroid function tests at follow-up post hospital discharge [82, 84, 86] Clinical, biochemical and ultrasound evidences of subacute thyroiditis during recovery phase [87–92] Low TSH and FT3 levels associated with normal/elevated FT4 [93]
Hypothalamus/Pituitary axis	SARS	Focal cell damage and reduction of TSH-positive, ACTH-positive and GH-positive cells by IHC [71] Increased number of PRL-, LH-e and FSH-positive cells by IHC [71] Detection of SARS-CoV-1 genome sequences in hypothalamus/pituitary cells from autoptic tissues by in situ Hybridization and RT-PCR [68–70]	83% of patients had central hypocortisolism with concomitant low or inappropriately normal ACTH levels [74] Increased PRL, LH, FSH serum levels in male patients [72, 73]
	COVID-19	Presence of SARS-CoV-2 in the cerebrospinal fluid of patients [75]	Decrease of GH and IGFBP-3 levels [76] 34% of patients displayed isolated low TSH values [82]
Adrenal Gland	SARS	Thrombosis and vasculitis in the adrenal vessels [65, 70] Hybridization in situ detected SARS-CoV-1 genome sequences in autoptic tissues [68]	
	COVID-19	Infiltration of CD3+ and CD8+ lymphocytes in different layers of cortex and in surrounding tissue [94] Small groups of proliferating cells with enlarged clear nuclei [94] Predominant vascular damage localized to the adrenals rather than the other organs [95, 96] Acute fibrinoid necrosis of adrenal arteriolae both in the parenchyma and capsule [96]. Focal inflammation [95] Adrenal parenchymal infarcts or thrombosis [95, 96]	Reports of acute bilateral adrenal hemorrhage and consequent acute adrenal failure [97, 98]
Ovary	SARS	No detection SARS-CoV-1 RNA polymerase by immunohistochemistry and in situ hybridization [69]	
	COVID-19	/	/
Testis	SARS	Extensive destruction of testicular germ cells [105, 106] Rare spermatozoa in the epithelium and lumen of seminiferous tubules [105, 106] Peritubular fibrosis [105, 106] Massive leukocyte infiltration and IgG presence [105, 106] Conflicting evidence about the presence of SARS-CoV-1 RNA in testicular cells by in situhybridization [69, 107]	Reduced testosterone levels in male patients [72, 73] Increased levels of LH and FSH in SARS males [72, 73]
	COVID-19	Sertoli cells: variable degree of swelling, vacuolation and cytoplasmic rarefaction, detachment from tubular basement membranes and sloughing into lumens of the intratubular cell mass [108] Reduced number of Leydig cells [108] Infiltrates of lymphocytes, macrophages and histiocytes in the interstitium [108–110] Thinning of seminiferous tubules with a significant high number of apoptotic cells and IgG inside [110] Oligozoospermia and significant increase of semen leucocyte number in 39.1% and 60.9% of COVID-19 patients, respectively [110] Conflicting evidences about the presence of SARS-CoV-2 RNA in testicular cells by RT-PCR [108, 110] Conflicting evidence about the presence of SARS-CoV-2 RNA in semen by RT-PCR [112–116]	Significant increase in serum LH, while T/LH and decrease of FSH/LH ratios [111] Not significant changes in serum testosterone levels between patients and control groups [111]

#### Effect of coronavirus on the hypothalamus/pituitary axis

Since 2002, the expression of ACE2 receptors in hypothalamus/pituitary [11–13] and the presence of neurological symptoms in subjects affected by SARS led to hypothesize that betacoronavirus infections may also affect central nervous system and, as a consequence, hypothalamus and pituitary. The main entrance to the central nervous system (CNS) for SARS-CoV-1 and SARS-CoV-2 is still uncertain and may be both indirect via bloodstream or direct via the cribriform plate. Moreover, several direct and indirect evidences suggest that betacoronavirus infection may exert a general depression of the hypothalamus/pituitary axis related to the burden of the infection and general hypoxia of the infected patients.

##### SARS-CoV-1 and hypothalamus/pituitary

In situ hybridization studies detected the expression of SARS-CoV-1 RNA polymerase gene in pituitary cells from autoptic tissues of SARS-CoV-1 patients [68, 69]. In 2005, Gu et al. found SARS genome sequences in the brains of 8 SARS autoptic cases by real-time RT-PCR. Hybridization studies localized these positive signals in cytoplasm of the neurons of cortex and hypothalamus [70]. In addition, an autoptic study performed in pituitary of five SARS patients with an age ranging 24–51 years indicated a reduction in TSH-positive, ACTH-positive and GH-positive cells and a concomitant focal cell damage. However, the reduction of ACTH-, TSH- and GH-positive pituitary cells described by Wei et al., could reflect a glucocorticoid-induced reduction of pituitary secretory granules [71]. On the other hand, PRL-positive, LH-positive and FSH-positive cells were increased in number and immunoreactivity. This last finding was in accordance with other studies describing increased levels of PRL, LH, FSH and reduced testosterone levels in SARS males [71–73]. In accordance with these morphological findings, studies aimed at evaluating endocrine function in SARS patients also found important abnormalities. A prospective study, performed in 61 SARS survivors, evaluated hormonal changes during 3 months after recovery. Patients with pre-existing endocrine disorders were excluded from the study and endocrine abnormalities were detected and treated up to one year after recovery from SARS. In this study, twenty-four (39.3%) patients displayed a variable degree of hypocortisolism even after normalization for age, sex and menopausal status. Twelve cases of the hypocortisolic cohort (83.3%) had unequivocal central hypocortisolism as evidenced by concomitant low or inappropriately normal ACTH levels [74].

##### SARS-CoV-2 and hypothalamus/pituitary

The first observation regarding SARS-CoV-2 was reported by Zhou et al., who found the presence of SARS-CoV-2 in the cerebrospinal fluid of COVID-19 patients, suggesting a SARS-CoV-2 spreading in the CNS [75]. Hence, it is reasonable to suppose that during the acute phases of the systemic inflammatory SARS-CoV-2 disease, the blood–brain barrier may become more permeable, thereby allowing the entry of the virus into the CNS, and its spread into the hypothalamus/pituitary. A functional study performed in 40 COVID-19 patients with non-severe symptoms matched to 54 healthy controls showed a significant decrease in GH and IGFBP-3 during hospitalization [76]. To better define the potential effects of COVID-19 on endocrine organs, it should be kept into account the burden of extrapulmonary micro-thrombosis commonly observed in COVID-19 patients, phaenomenon that was less frequent in SARS [67, 77]. Hence, this specific pathogenetic effect of COVID-19 may affect highly vascularized organs, such as the endocrine glands, and in particular those with a very dense vascular network including pituitary. Further investigations, however, are necessary to confirm a hypothalamus/pituitary involvement during COVID-19 infection.

#### Effect of coronavirus on thyroid

Several evidences suggest that both SARS-CoV-1 and SARS-CoV-2 may have an impact on thyroid tissue and function, although with both overlapping and different effects. As already mentioned for the effect of coronavirus on hypothalamus/pituitary axis, a general nonspecific “low T3 syndrome effect” may also play an important role.

##### SARS-CoV-1 and thyroid

A study performed in 2006 evaluated the pathological features of thyroid tissue in five SARS-Cov-positive subjects. Autopsies displayed a derangement of the follicular architecture with a various degree of damaged follicular cells and an increased interfollicular fibrosis [78]. Moreover, calcitonin-positive cells were completely absent in SARS patients, at variance with controls [78]. Tunel assay showed high level of apoptosis in all SARS patients, but not in controls, in both follicular epithelium and in interfollicular region [78]. Further studies on thyroid function reported that TSH, FT3 and FT4 levels in SARS patients were significantly lower than control group. In those patients, FT3 level was inversely related to the severity of the disease. In particular, in SARS patients, serum FT3 and FT4 levels decreased by 94% and 46%, respectively, during the acute phase and in 90% and 38% during the recovery phase [79].

The large extent of morphological injury and the quantity of apoptotic follicular cells provide an explanation for the decreased serum T3 and T4 levels in patients with SARS [79]. In contrast, the reduced TSH level reported in patients with SARS cannot be explained by the destruction of follicular epithelium, as low T3 and T4 levels would result in higher TSH levels.

According to Leow et al. [74], SARS would cause a central hypothyroidism by inducing hypophysitis as suggested by the central hypocortisolism observed in several SARS patients. In addition, a SARS effect on hypothalamic TRH producing cells cannot be also excluded. Such possible effect on the hypothalamus is consistent with the presence of oedema and neuronal degeneration together with the identification of viral genome sequences in the hypothalamus and cortex of the brain of patients with SARS [70]. Another hypothesis to explain such a thyroid hormonal setting is the low T3 syndrome due to both the severe lung infection, with the consequent hypoxemia, and the concomitant high-dose administration of glucocorticoids [80, 81].

##### SARS-CoV-2 and thyroid

A recent autoptic study examined pathology features of thyroid gland in three patients who died by SARS-CoV-2. The authors observed no abnormalities in the thyroid follicular cells, while they found interstitial lymphocytic infiltration. Immunohistochemistry and PCR analysis were not able to detect SARS-CoV-2 in thyroid tissue [67]. These findings are different to those reported in SARS-CoV-1 individuals, although the general patient clinical conditions in both studies were similar. With respect to thyroid function changes related to SARS-CoV-2 infection, recent papers substantially describe three different patterns of biochemical changes, only partially overlapping with those observed in SARS-CoV-1 patients. In particular, a retrospective study by Chen et al. analysed a group of 50 SARS-CoV-2 patients matched with non-COVID-19 pneumonia patients with a similar degree of disease severity. They found that the degree of the decrease in TSH and TT3 levels was positively related to the severity of COVID-19 infection. All enrolled patients had no previous known thyroid disease and no medical record influencing thyroid function. After recovery, no significant differences in TSH, TT3, TT4, FT3 and FT4 levels were found between the COVID-19 and control groups [82].

Another group performed a study in 274 patients with SARS-CoV-2 and found that TSH and FT3 concentrations were significantly lower in patients who died (n.113) than those who recovered (n.161), while FT4 levels were not statistically different. In all these patients, mortality correlated with the severity of thyroid hormone changes, while pre-existent thyroid diseases were not recorded [83]. This correlation was confirmed by further studies [84, 85], in particular Gao et al. grouped 100 patients into non-severely ill patients, survivors and non-survivors displaying mean FT3 values of 4.40, 3.73 and 2.76 pmol/L, respectively. Hence, FT3 levels were significantly lower in patients with severe COVID-19 disease and FT3 levels lower than 3.10 pmol/L predicted morality independently from all other causes [84]. Moreover, a study was performed in 40 COVID-19 patients with non-severe symptoms, matched to 54 healthy subjects by age and gender; serum samples were collected at 1st, 4th, 7th and 10th day of hospitalization and, compared to controls. Patients showed a reduction in TSH and FT3 and an increase in PTH levels with a concomitant reduction of vitamin D, calcium and albumin [76]. The reduction in TSH and FT3 levels in COVID-19 patients, similar to that observed in SARS patients, may be attributed to non-thyroidal illness syndrome or euthyroid sick syndrome [80, 81], induced by both hypoxemia and glucocorticoid treatment, as observed by Khoo et al. on a large cohort of patients with mild reductions of TSH and FT4 in admission to hospital and normalization of thyroid function tests at follow-up post discharge [86]. However, Chen et al., hypothesized the possibility of a selective transient pituitary deregulation, due to either the direct cytotoxic effect of the virus at the pituitary level or an indirect effect via the activation of proinflammatory cytokines. This hypothesis was supported by the observation that 34% (17/50) of the patients displayed isolated low TSH values during course of COVID-19 infection [82]. On the other hand, this idea does not fit with concomitant normal FT4 levels.

Other studies showed a direct damage of thyroid tissue in response to COVID-19 infection describing some patients with neck pain radiated to the jaw and concomitant asthenia. Laboratory tests performed in this cohort displayed high levels of both FT4 and FT3, undetectable serum levels of TSH. Neck ultrasound was able to detect multiple diffuse hypoechoic areas with decreased vascularity. Taken together, all these features were suggestive of a typical subacute thyroiditis [87–91]. All necessary tests were performed in each patient to confirm the hypothesis of subacute thyroiditis and exclude other causes thyrotoxicosis. Interestingly, the symptoms of thyroiditis appeared after the resolution of the respiratory symptoms and negativity of swab test, with the exception of two cases developing subacute thyroiditis concomitantly to COVID-19 infection. The available data indicated that all the patients had a mild COVID-19 infection and none of them displayed a positive second swab test during the occurrence of thyroiditis. Moreover, all the patients were young women without any evidence of previous thyroid disease.

In addition, Lania et al., retrospectively evaluated thyroid hormones and serum interleukin-6 (IL-6) levels in 287 COVID-19 patients, hospitalized in non-intensive care units. They found that 58 patients (20.2%) displayed thyrotoxicosis (overt in 31 cases), 15 (5.2%) hypothyroidism (overt in 2 cases), 214 (74.6%) euthyroidism. Multivariate logistic regression analysis revealed that thyrotoxicosis was positively related to higher IL-6 levels (odds ratio 3.25, 95% confidence interval 1.97–5.36; *p* < 0.001) [92].

Different mechanisms may explain all these observations: (1) high ACE2 and TMPRSS2 expression in thyroid [11, 12] may facilitate the COVID-19 attack and cytolysis, thereby triggering an autonomous inflammatory process in predisposed subjects, which progresses after the resolution of the COVID-19 infection; (2) systemic immune activation in response to SARS-CoV-2 infection may cause thyroid damage with thyrotoxicosis.

Finally, an additional mechanism may be postulated by the observation of Muller et al. They compared 85 COVID-19 patients admitted to high intensity of care units (HICUs) in 2020, to 78 admitted to the same HICUs in 2019 with a similar clinical setting but negative for SARS-CoV-2: patients with a known history of thyroid disease were excluded. In these patients, thyroid function was assessed within 2 days of hospital admittance. Interestingly, 15% of COVID-19 patients displayed thyrotoxicosis compared to 1%, in the control group. The Sex of patients with thyrotoxicosis and COVID-19 infection was predominantly male and low TSH and FT3 level were associated with normal/elevated FT4 level [93]. Hence, authors speculated that these patients were affected by a combination of thyrotoxicosis and non-thyroidal illness syndrome.

#### Effect of coronavirus on adrenal gland

Some evidences, including the expression of ACE2 receptor in adrenal glands, suggest a possible relationship between SARS or COVID-19 infection and primary adrenal insufficiency. Moreover, several direct and indirect evidences suggest that betacoronavirus infection may induce a central adrenal insufficiency related to the burden of the infection, general hypoxia of the infected patients and glucocorticoid therapy.

##### SARS-CoV-1 and adrenals

ACE2 receptor expression and the presence of SARS-CoV-1 RNA were detected in adrenal gland [11–18]. Autoptic analysis in SARS-positive subjects revealed degeneration and necrosis of the adrenal cortical cells due to either cytopathic effect of the virus or to vasculitis/thrombosis of the adrenal vessels [65, 70]. However, no clinical studies are available to date demonstrating primary adrenal insufficiency related to SARS-CoV-1 infection.

##### SARS-CoV-2 and adrenals

Zinserling et al., conducted a detailed autoptic study in 10 patients deceased from COVID-19, describing two types of adrenal lesions. The first one was an immune cell infiltration of different layers of the cortex and surrounding tissue. Immunohistochemistry was able to characterize infiltrating cells as CD3+ and CD8+. The second one was characterized by the presence of small groups of proliferating cells with enlarged clear nuclei [94]. Such changes were similar to those observed in the lungs and were considered to be a direct effect of SARS-CoV-2. Hence, patients with COVID-19 infection may be susceptible to corticosteroid insufficiency (CIRCI) due to both direct viral adrenal cell damage and adrenal inflammatory/autoimmune processes. The variability of microscopic alterations induced by SARS-CoV-2 on human adrenals was confirmed by a recent study from Freire Santana et al. They performed autoptic analysis in 28 COVID patients and observed microscopic lesions in the adrenal glands of 12 out 28 patients (46%): seven showed ischemic necrosis; four cortical lipid degeneration; two haemorrhage; one unspecific focal adrenalitis; one vascular thrombosis; three focal inflammation along with the other findings [95]. However, further studies will be required to prove the presence of SARS-CoV-2 in adrenal tissue and define the mechanisms of adrenal degeneration and loss of function. Iuga et al. conducted another study in five patients deceased from COVID-19 and found a predominant vascular damage localized to the adrenals rather than the other organs. Microscopic examination evidenced acute fibrinoid necrosis of adrenal arteriolae both in the parenchyma and capsule, with some aspects of subendothelial vacuolization and apoptotic debris, without any significant inflammation, adrenal parenchymal infarcts or thrombosis. Many of the vessels observed displayed either necrosis or apoptosis. It is unclear whether the adrenal vasculopathy is due to hypoxia, abnormal vascular reaction, direct viral cytopathic effect, immune-mediated injury or a combination of events [96].

Finally, two case reports of COVID-19 patients with bilateral acute adrenal hemorrhage were described in the literature. In particular, one case is a 66-year-old woman, hospitalized with fever, dyspnea, abdominal pain, vomiting and nausea with simultaneous diagnosis of COVID-19. Chest X-ray confirmed atypical pneumonia due to COVID-19, while abdomen TC displayed the enlarged adrenal glands, haziness of peri-adrenal fat and thrombosis of left renal vein. Serum cortisol level was very low and unresponsive to 250 µg intravenous Cosynotropin. Treatment for acute adrenal failure was started, with subsequent stabilization of clinical conditions and symptom resolution. According to patient’s history of recurrent abortions, the presence of antiphospholipid antibody syndrome (APLS) was also suspected and confirmed by specific antibody assays. Hence, authors hypothesized that the combination of both COVID-19 infection and APLS was responsible for adrenal failure [97]. Another case is a 70-year-old man, with a history of psoriasis, hospitalized with persistent lower back pain resistant to medical treatment. Fifteen days before pain onset, he had fever, chills, and asthenia and before hospital admission, fatigue, anorexia and nausea. During hospitalization, chest CT scan evidenced bilateral bronchopneumonia, compatible to COVID-19 infection. Abdomen CT scan showed increased size and blurring of both adrenals suggestive for acute bilateral adrenal hemorrhage (BAH). The patient was positive for COVID-19 IgG and IgM. Both basal cortisol and stimulated confirmed the diagnosis of adrenal insufficiency [98] and intravenous corticosteroid treatment was started followed by oral therapy with subsequent symptom resolution. In these two cases, the presence of underlying autoimmune disease may predispose COVID-19 patients to develop coagulation disorders and disseminated intravascular coagulation (DIC) and thrombosis with subsequent hemorrhage in the most vascularized organs [99]. Hence, in COVID-19 patients with fever, nausea, malaise, physicians should check for BAH possibility by functional and imaging studies.

#### Effect of coronavirus on ovary

Several evidences are present about susceptibility of the ovary to the effect of SARS-CoV-1 and SARS-CoV-2 infection. Many studies are available focusing mainly on the expression of ACE2 receptor in the ovary, as an indirect evidence of a potential damage by SARS-CoV-1 and SARS-CoV-2. In particular, ACE2 mRNA transcripts were detected in ovaries from both fertile and postmenopausal women [100] and these findings are confirmed by studies on gene databases that describe a significant ACE2 receptor expression in the ovaries [11, 101]. Goad et al., found a very low expression of ACE2 receptor in approximately 5% of stroma and perivascular cells in the ovarian cortex. They did not observe any concomitant expression of TMPRSS2 in any ovary cell type and a certain degree of CTSB and CTSL expression. However, they did not observe any co-expression of ACE2/CTSB or ACE2/CTSL. Since ACE2 receptor requires the co-expression of protease TMPRSS2 or CTSB / L to facilitate virus entry into the host cell by priming the S protein on its surface, these data suggest that sensitivity of the ovary to SARS-CoV-1 and SARS-CoV-2 infection and damage may be low [102].

##### SARS-CoV-1 and ovary

Evidences regarding the involvement of ovary in SARS-CoV-1 infection are scanty. Immunohistochemistry and in situ hybridization studies by Ding et al. were not able to detect SARS-CoV-1 RNA polymerase in the ovary of four patients who died by SARS [69].

##### SARS-CoV-2 and ovary

Evidences regarding the involvement of ovary in SARS-CoV-2 infection are missing. Hence, post-mortem pathological studies on consistent series are necessary to clarify any possibility of COVID-19 infection in the ovary and its potential effect on female fertility.

#### Effect of coronavirus on testis

ACE2 receptor expression is very high in Spermatogonia, Sertoli and Leydig cells [11, 12, 103, 104] and it is reasonable to suppose that testis may be heavily infected by both SARS-Cov-1 and SARS-CoV-2.

##### SARS-CoV-1 and testis

A series of autoptic studies indicated that orchitis is a SARS complication, with a pathological aspect of extensive destruction of testicular germ cells, rare spermatozoa in the seminiferous epithelium and in the lumen and thickening of the membrane, associated with peritubular fibrosis. These features are attributed to leukocyte infiltration, vascular congestion and the presence of IgG at both tubular and interstitial levels [70, 105, 106]. However, there are some conflicting evidences about the presence of SARS-CoV-1 RNA in testicular cells [69, 107].

##### SARS-CoV-2 and testis

Yang et al., performed autoptic examination of testes from 11 COVID-19 patients by light, electron microscopy, immunohistochemistry and RT-PCR. The mean age was 65 years (range 42–87 years). The mean disease duration (from onset to death) was 42 days (range 23–75 days). From microscopy, Sertoli cells displayed a variable degree of swelling, vacuolation and cytoplasmic rarefaction, detachment from tubular basement membranes and sloughing into lumens of the intratubular cell mass. The mean number of Leydig cells in COVID-19 testes was significantly lower than in the control group (2.2 vs 7.8, *p* < 0.001) and infiltrates of T lymphocytes and histiocytes were present in the interstitium. Transmission electron microscopy performed in 3 out 12 cases was not able to identify SARS-CoV-2 viral particles, while RT-PCR was able to detect the virus in one case [108]. This lymphocytic and macrophage infiltration was confirmed by more recent studies in six autoptic samples by Achua et al., along finding of normal spermatogenesis in 50% of the samples and various abnormalities of spermatogenesis in the remaining 50% [109]. Moreover, Li et al. evaluated six testicular and epididymal autoptic specimens and found interstitial edema, congestion, red blood cell exudation in testes/epididymides and thinning of seminiferous tubules, with an increased concentration of CD3+ and CD68+ in the interstitium. The significant high number of apoptotic cells within seminiferous tubules and the presence of IgG suggested impaired spermatogenesis in COVID-19 patients. Hence, they also evaluated semen from 23 COVID-19 patients and found that 39.1% (*n* = 9) had oligozoospermia and 60.9% (*n* = 14) had a significant increase in leucocyte number. Increased seminal level of IL-6, TNF-a and MCP-1 compared to controls was also observed. All semen samples were negative for SARS-CoV-2 RNA and the patients had no history of infertility or steroid treatment [110]. These findings are comparable with those obtained with SARS-CoV-1 patients. Interestingly, a study performed in 81 COVID-19 adult male patients and 100 age-matched healthy controls evidenced a significant increase in serum luteinizing hormone (LH), while T/LH and FSH/LH ratios were dramatically decreased. Serum testosterone levels did not significantly change between COVID-19 patients and control groups. Elevated serum LH and decreased T/LH ratio are clinical hallmark of primary hypogonadism, suggesting testicular damage and Leydig cells involvement [111]. However, the long-term testicular effects of COVID-19 are not known to date. Based on these evidences, suggesting extensive testicular involvement by SARS-CoV-2, the possibility of virus relapse with seminal fluid, with potential effects on transmission, fertility and cryopreservation is under debate. Pan et al., were not able to detect SARS-CoV-2 in the semen collected from 34 COVID-19 patients with mild–moderate symptoms in a period between 8 and 75 days (median 31 days) after COVID-19 diagnosis, despite 19% of them complained about scrotal discomfort at the time of COVID-19 diagnosis [112]. In accordance with these data, Song et al., were not able to detect SARS-CoV-2 RNA in the semen from 12 patients with asymptomatic/mild COVID-19 disease in Wuhan in a period between 14 and 42 days after COVID-19 diagnosis. Moreover, the authors were not able to detect COVID-19 RNA in testicular tissue from deceased subjects [113]. A case report showed that a 31-year-old man recovering a mild form of COVID-19 had no detectable virus in his ejaculate within fifteen days from the onset of the disease [114].

Another study compared semen samples from 18 COVID-19 male patients 8–54 days after the absence of symptoms, 14 control subjects, and 2 patients with an active COVID-19 infection. No viral RNA was detected by RT-PCR in the semen. Interestingly, subjects with a moderate infection showed an impairment of sperm quality (sperm concentration, progressive motility, total number of complete motility) compared with men recovered from a mild infection and the control group [115].

To date, only one study by Li et al. was able to detect the virus in 6/38 semen samples collected from both acute and recovering COVID-19 patients [116] (Table 4). While this finding appears in contrast with the previous investigations, it needs to be cautiously interpreted. First, this study was performed in a dedicated COVID-19 hospital, where the most severe cases of COVID-19 were admitted. Hence, a more severe disease is concomitant with a higher blood viral titer and a higher chance to spread to other organs and body fluids including the semen. In particular, the blood–testis barrier (BTB) is permeable to viruses, particularly in the case of systemic or local inflammation and viraemia [117]. Moreover, in a COVID-19 dedicated hospital, there is higher probability of viral spread in the environment, false-positive results could be obtained because of contamination with respiratory droplets. However, available data are too scanty to define this issue and studies performed in larger cohorts of currently infected subjects are needed. This topic is crucial for the safety of sperm cryopreservation in liquid nitrogen and for assisted reproduction techniques [118].

**Table 4:**
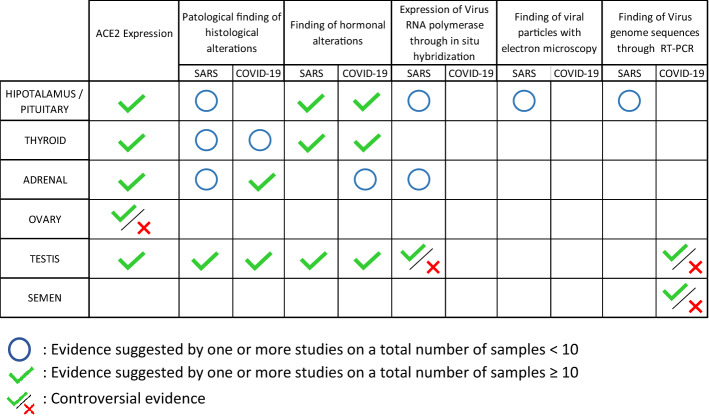
Schematic summary of the results

Finally, it would be interesting to evaluate the presence of viral particles in the first days of COVID-19 infection, when patients are asymptomatic.

## Limits

The major limitation of this review is the limited number of studies performed on patients with SARS and COVID-19 that are focused on the endocrine system, because the very recent outbreak of the pandemic. Furthermore, studies are mostly performed in small groups of either patients or autoptic samples. Finally, available studies are heterogeneous in terms of study design, participants and outcomes which makes difficult comparison of data.

## Conclusion

There are many common elements between SARS-CoV-2 and other betacoronaviruses that have previously caused outbreaks around the world, most notably SARS-CoV-1. Both viruses recognize the ACE2 protein as a target of cellular infection and this protein is widely expressed in endocrine organs. Several reports are available indicating that SARS-CoV-1 and SARS-CoV-2 may affect the endocrine glands and their dense vascularity. It is, therefore, reasonable to suppose that with the spread worldwide of COVID-19, that damage to the endocrine system may emerge more frequently in the future. This review suggests that possible hormonal alterations in patients with COVID-19 should be evaluated both in the acute phase of infection and in recovery to rapidly identify acute- or late-onset endocrine complications critical to patient’s prognosis and well-being post COVID. Therefore, further prospective studies in patients with COVID-19 are needed to improve the management of this pandemic disease. Data about the effect of COVID-19 infection on thyroid, pituitary and adrenals are often based upon the observation of small series, but are suggestive of a real effect, while the gonad’s involvement remains largely unexplored. Moreover, some data on endocrine effect of COVID-19 are not mechanistic and mostly conjectural and factual up to date. An emerging issue is the hormonal regulation of protein on cell surface that facilitate viral entry and spread. Although the evidences of such regulation are progressively establishing, the possible therapeutic implication of a hormonal manipulation to influence disease severity is scanty and limited mainly to glucocorticoids. Finally, validated conclusions must be drawn based on larger studies and endocrinologists, however, need to be aware of these possibilities in clinical practice, especially while dealing with COVID-19 survivors.
